# Asymetric Event-Related Potential Priming Effects Between English Letters and American Sign Language Fingerspelling Fonts

**DOI:** 10.1162/nol_a_00104

**Published:** 2023-06-13

**Authors:** Zed Sevcikova Sehyr, Katherine J. Midgley, Karen Emmorey, Phillip J. Holcomb

**Affiliations:** San Diego State University Research Foundation, San Diego State University, San Diego, CA, USA; School of Speech, Language, and Hearing Sciences, San Diego State University, San Diego, CA, USA; Department of Psychology, San Diego State University, San Diego, CA, USA

**Keywords:** American Sign Language, ERPs, fingerspelling, orthography

## Abstract

Letter recognition plays an important role in reading and follows different phases of processing, from early visual feature detection to the access of abstract letter representations. Deaf ASL–English bilinguals experience orthography in two forms: English letters and fingerspelling. However, the neurobiological nature of fingerspelling representations, and the relationship between the two orthographies, remains unexplored. We examined the temporal dynamics of single English letter and ASL fingerspelling font processing in an unmasked priming paradigm with centrally presented targets for 200 ms preceded by 100 ms primes. Event-related brain potentials were recorded while participants performed a probe detection task. Experiment 1 examined English letter-to-letter priming in deaf signers and hearing non-signers. We found that English letter recognition is similar for deaf and hearing readers, extending previous findings with hearing readers to unmasked presentations. Experiment 2 examined priming effects between English letters and ASL fingerspelling fonts in deaf signers only. We found that fingerspelling fonts primed both fingerspelling fonts and English letters, but English letters did not prime fingerspelling fonts, indicating a priming asymmetry between letters and fingerspelling fonts. We also found an N400-like priming effect when the primes were fingerspelling fonts which might reflect strategic access to the lexical names of letters. The studies suggest that deaf ASL–English bilinguals process English letters and ASL fingerspelling differently and that the two systems may have distinct neural representations. However, the fact that fingerspelling fonts can prime English letters suggests that the two orthographies may share abstract representations to some extent.

## INTRODUCTION

Orthography is traditionally defined as a method of representing continuous speech sounds by written symbols (i.e., alphabetic letters such as the Roman alphabet). In American Sign Language (ASL), orthography can also be represented through ASL fingerspelling. Fingerspelling is a manual, typically non-written symbol system in which each letter of an alphabet is represented by a distinct handshape. Although fingerspelling handshapes are based on English orthography, the nature and the possible interrelationship between the representational systems underlying the written and fingerspelled handshape systems remain unexplored. Here, we first examined how deaf and hearing adults represent and process single English letters from a Roman alphabet, using an unmasked priming paradigm. Using the same type of priming paradigm, we then assessed how deaf adult signers process single ASL fingerspelling handshapes (represented via fingerspelling fonts) and whether the two representational systems, letters and fingerspelling handshapes, prime each other. Charting the time course of visual letter and fingerspelling handshape recognition allows us to characterize the nature of orthographic codes in deaf readers, which has theoretical importance for understanding the mechanisms of the reading system as well as practical implications for developing reading interventions for deaf people.

The relationship between fingerspelling and print reading skills in deaf individuals has been of growing interest. Fingerspelling is used relatively frequently in ASL (Padden, 2006) in both home and educational contexts and has been argued to play a beneficial role in connecting signs to printed words through common instructional methods, such as “chaining,” which involves making a series of associations between fingerspelled words and printed words by first fingerspelling the target word, pointing to the printed word, and then fingerspelling it again (Humphries & MacDougall, 2000; Padden & Ramsey, 2000). Deaf children learn to identify fingerspelling handshapes and map them to English letters and words (Chamberlain & Mayberry, 2000; Haptonstall-Nykaza & Schick, 2007; Hirsh-Pasek, 1987; Padden & Ramsey, 2000). This segmentation and mapping process is hypothesized to assist deaf children in learning to analyze and segment printed words (Emmorey & Petrich, 2012; Stone et al., 2015) and may lead to better word decoding and recognition (Haptonstall-Nykaza & Schick, 2007; Hirsh-Pasek, 1987). Many studies, including longitudinal and intervention studies, have now shown that fingerspelling might mediate initial orthographic development and play an important role in the construction of orthographical or phonological representations of printed words and ultimately contribute to skilled reading comprehension (Haptonstall-Nykaza & Schick, 2007; Miller et al., 2021; Ormel et al., 2022; Padden & Ramsey, 1998, 2000; Sehyr & Emmorey, 2022; Stone et al., 2015).

Despite compelling evidence for the association between fingerspelling and printed words, the relationship between these two highly distinct yet seemingly functionally connected orthographic systems remains unexplored. In this article, we aim to tackle the question of whether ASL fingerspelling handshapes that come to represent the individual English letters share underlying abstract representations. Moreover, orthographic recognition at the single letter level has not been examined in deaf readers. Collective knowledge about orthographic representations in deaf readers comes from studies that focused on recognition of visually presented whole words. At the word level, skilled deaf readers exhibit high selectivity to orthography in both the left visual word form area (VWFA) and in the inferior frontal gyrus (Glezer et al., 2018), similarly to hearing readers. In contrast to hearing readers, deaf readers tend to develop more bilaterally tuned neural representations to written words, perhaps due to reduced access to phonological representations during word recognition (Emmorey et al., 2017; Glezer et al., 2018; Sehyr et al., 2020). Deaf skilled readers may instead develop stronger connections between orthography and lexical-semantic representations (Emmorey & Lee, 2021; Gutierrez-Sigut et al., 2019; Sehyr & Emmorey, 2022). Further, comprehension and production of fingerspelled words engage the VWFA similarly to printed words, suggesting that this neural region may support amodal orthographic representations (Emmorey et al., 2015, 2016; Waters et al., 2007). Therefore, an open question is whether experience with fingerspelling might alter the neural responses to letters.

The reported differences in orthographic processing compel us to assess whether differences between deaf and hearing readers might propagate to single letter processing. The processing of whole words is influenced by semantic, phonological, and orthographic properties that can be sensitive to individual differences in reading skills and exposure. For example, Gutierrez-Sigut et al. (2019) provide event-related potential (ERP) evidence that more skilled deaf readers have a stronger connection between orthographic and lexical-semantic levels of processing. It is possible that top-down lexical-semantic representations might modulate letter-in-word processing in ways that impact orthographic representations at the level of individual letters. Letter processing, however, becomes automatized in the early stages of reading development and may be considerably less influenced by linguistic experiences or exposure than words (Flowers et al., 2004). Fingerspelling, or fingerspelling fonts that represent fingerspelled letters, are not used for text reading and may be less likely to become automatically processed in the way that printed letters do. Studying the processing of single letters and fingerspelling fonts without lexical context can help to determine whether these orthographic stimuli access shared abstract representations. If shared orthographic representations between letters and handshape fonts exist, we would observe priming effects prior to lexical-semantic processing (i.e., prior to an N400 component).

Letter recognition is hypothesized to consist of several stages of processing, from early visual processes during which the physical sub-letter properties (e.g., lines or curvature of fonts) and case-specific higher-level representation (e.g., case-dependent representations) are extracted, to activation of abstract case-independent representations (e.g., the nominal identity of letters) in later stages of recognition. These stages have been associated with different ERP components (see Petit et al., 2006). The authors measured ERPs to single English letter targets briefly preceded by related (repeated) or unrelated masked letter primes in hearing readers and found evidence for processing differences in all three stages.

Relatively little research has aimed at elucidating the nature of orthographic representations and the neural basis of single letter processing in deaf readers. In Experiment 1, we examined the time course of single letter processing in hearing and deaf readers using a single letter, unmasked, repetition priming paradigm adapted from Petit et al. (2006). Previous studies found differences between deaf and hearing readers in orthographic processing at the word level (e.g., see Emmorey et al., 2017; Glezer et al., 2018; Sehyr et al., 2020). Therefore, the goal of this study was to establish whether group differences also occur at the level of letter processing. This knowledge served as a basis for interpreting the results of the second experiment with deaf readers; if we found differences between letter and fingerspelling processing, we first had to establish that this result was related to the stimulus processing and not due to general changes in how deaf readers process letters. Additionally, evidence for letter-to-letter priming as found in Petit et al. would allow us to extend previous findings to a supraliminal priming paradigm and to readers who are deaf.

In Experiment 2, we sought to answer a basic question: At what level of processing do print and fingerspelling handshapes share common underlying neural representations? The goal was to examine whether priming effects differ for English letters, fingerspelling fonts, and across the letter and fingerspelling systems (“domains” from here on) in deaf participants. Here we sought to determine a time window in a supraliminal priming paradigm that would reflect the abstract processing of letters observed by Petit et al. (2006). This time window would have similar directionality (related primes showing less positivity than unrelated primes) and a similar widespread scalp distribution. Note that typical fingerspelling production includes transitional movements between handshapes and this visually dynamic signal would likely yield highly distinct brain responses compared to those elicited by static English letters. Fingerspelling handshapes are, however, easily recognized from static line drawings (except J and Z, which require wrist notation and were excluded from this study). Fingerspelling font can be used to legibly represent individual handshapes in the manual fingerspelling alphabet and may be used in educational settings. In this study, we used static fingerspelling fonts instead of dynamic fingerspelling or realistic photographs to better match letters and fingerspelling in visual features (e.g., static lines, curves) to allow for a more direct comparison of the elicited waveforms to these stimuli. Previous research has also shown that people extract motion information or reconstruct three-dimensional representations from still images. For example, static line drawings can trigger three-dimensional surface representations (Barrow & Tenenbaum, 1981; Sayim & Cavanagh, 2011) and activate motion-sensitive cortex (Proverbio et al., 2009). Based on this evidence, we assumed that deaf signers would access dynamic fingerspelling representations from the line drawings used to create the fingerspelling fonts. We also verified that deaf participants were able to accurately recognize the fingerspelling fonts by administering a fingerspelling font labeling task to all deaf participants before Experiment 2, in which they performed at near-ceiling accuracy.

In both experiments, we recorded ERPs from the participants’ scalp while they performed a probe detection task. To facilitate a comparison of the time course and stages of orthographic processing with those reported by Petit et al. (2006), we employed the same basic priming paradigm used in their study with the exception that the forward and backward masks were not included. This design renders the prime stimuli supraliminal but keeps the timing of prime and target stimuli the same as in Petit et al. (i.e., the same 100 ms stimulus onset asynchrony). We made this change because it is not clear what the comparable masking parameters would be for English and fingerspelled stimuli, making any cross-category comparisons of priming problematic. Relatedly, because the targets immediately followed the primes, we slightly increased the size of the targets to perceptually distinguish them from the primes. Piloting the stimulus presentations revealed that this modification worked well for the letter pairs, but for the fingerspelling font pairs, this led to a perceived smooth forward motion effect. Therefore, we reversed the handedness of the handshape font when the prime and target were identical to avoid this perceptual effect. Handedness in ASL is not linguistically distinctive and depends on an individual’s hand preference. ASL fingerspelling is one-handed, and ASL signers typically use their dominant hand to fingerspell; however, signers do sometimes fingerspell with their non-dominant hand (e.g., to emphasize a contrast between two items or topics) and recognition of left-handed fingerspelling is not uncommon. Anecdotally, signers report not noticing whether a signer is left- or right-handed, and left-handed signing and fingerspelling is easily understood. This is further supported by prior research findings that ASL signers’ lexical decisions are not influenced by sign handedness, suggesting that signers develop representations that are less bound to the hand with which they are performed (Corina & Gutierrez, 2016). Thus, we did not expect the handedness manipulation to strongly influence handshape font processing during the critical time epochs. By administering the fingerspelling font labeling task prior to Experiment 2, we confirmed that participants were able to easily recognize both left- and right-handed fonts with at ceiling or near ceiling accuracy. Further, as we report in the results section, we found no difference in left- and right-hand probe detection, suggesting no difference in processing.

In Experiment 1, we expected that for hearing readers, English letter targets preceded by related English letter primes (i.e., identical letters) would elicit less positivity in the early stages of processing than targets preceded by unrelated (different letter) primes and that this effect would have a widespread distribution, based on the Petit et al. (2006) findings. However, we expected that our results may have a different timing than in Petit et al. because we used a supraliminal priming paradigm with a longer prime duration. We also did not analyze the initial feature/case sensitive stages of letter processing that were reported by Petit et al. (2006) between 120 and 220 ms post-stimulus onset because we did not include letter case as a condition in our paradigm as there is no upper or lower case for fingerspelling fonts. Therefore, we used visual inspection of our data to determine the time window congruous with Petit et al.’s findings for the direction and scalp distribution of abstract letter recognition. This allowed for the possibility that priming effects in an unmasked priming context might onset earlier or last longer compared to the masked presentation in Petit et al. (2006). In other words, we were interested in examining the waveforms during the stage of processing of abstract letter representations for which Petit et al. reported widespread effects between 220 and 300 ms. Additionally, waveform differences beyond 300 ms post-stimulus onset have not been typically reported in masked letter priming studies, including Petit et al. However, we selected a later time window (300–500 ms) as we expected that priming may occur in later stages due to the participants’ supraliminal awareness of both the prime and target.

Overall, we expected that deaf and hearing participants would pattern similarly in both early and later stages of letter processing. If so, this would suggest that the deaf readers letter recognition is not impacted by the experience of deafness or knowledge of fingerspelling. Alternatively, deaf participants might exhibit distinct priming effects or scalp distribution patterns of priming effects to letters because differences in neural-behavioral profiles between deaf and hearing readers for whole word recognition have been reported (Emmorey et al., 2017; Meade et al., 2020; Sehyr et al., 2020; Winsler et al., 2023). These patterns would suggest that deaf people develop distinct neural patterns in letter recognition, perhaps due to their unique sensory-perceptual and/or linguistic experience.

In Experiment 2, for deaf readers, we expected that within each orthographic domain—letters or fingerspelling fonts—the related prime-target pairs would elicit priming effects (i.e., less positivity) than unrelated pairs in both time windows. Importantly, if letters and fingerspelling fonts activate the same abstract representations, we should also observe priming effects for cross-domain presentations in the time window previously associated with an abstract level of processing. Failure to find cross-domain priming in the presence of within-domain priming would suggest that the two orthographic systems have distinct and non-overlapping neural representations. Finally, we did not expect priming between letters and fingerspelling in the initial stages because those effects tend to be related to feature/case processing. This is because English letters and fingerspelling fonts are distinct. Thus we looked for priming effects in the later stages of processing that are associated with abstract representation processing.

## EXPERIMENT 1: ENGLISH LETTER PRIMING IN DEAF SIGNERS AND HEARING NON-SIGNERS

### Method

#### Participants

Fifty-two adults were recruited to participate in this experiment. Twenty-six were monolingual hearing English speakers and 26 were severely to profoundly deaf bimodal bilinguals (ASL–English). Two participants from each group were excluded for high artifact rejection rates (>17% rejection in at least one condition). All participants reported having normal or corrected to normal vision, no history of neurological dysfunction or language disorders, and were not taking any medications that would affect brain function. They provided written informed consent in accordance with the Institutional Review Board at San Diego State University, which approved the study. In total, 24 deaf participants (13 female, *M* age = 35, *SD* = 8) and 24 hearing participants (16 female, *M* age = 29, *SD* = 9) were included in the analyses. The deaf participants were either native ASL signers who were born into deaf signing families and exposed to ASL from birth (*N* = 9) or were exposed to ASL prior to age 8 (*N* = 15; average age of ASL exposure = 30 months). All deaf participants reported ASL as their primary means of communication. Four deaf signers were left-handed, and no hearing participants were left-handed. Two deaf participants had a cochlear implant (CI); one participant received a CI at age 4 and stopped using it at age 22 years, and the other participant received a CI at age 20 and used it about 60% of the time at the time of the study. (The pattern of results remained the same if the two participants with CIs were excluded from the analyses.)

In addition, all participants completed a standardized test of spelling, the Spelling Recognition Test (Andrews & Hersch, 2010) as an offline measure of receptive orthographic skills. The test contains 88 items, half correctly spelled and half misspelled. Misspellings change one to three letters of the word and often preserve the pronunciation of the base word (e.g., *addmission*, *seperate*). Participants were instructed to circle items they thought were incorrectly spelled. The total score was the number of correctly classified items, both hits and correct rejections. The groups were both skilled spellers and did not statistically differ on spelling; deaf participants scored 75 (*SD* = 9.7) and hearing participants scored 76 (*SD* = 7.5), *p* = 0.679.

#### Stimuli

Twenty-three single English letters were used as the critical target stimuli in Experiment 1. The letters J, Z, and Y were excluded; the letters J and Z were excluded to keep consistent with stimuli for Experiment 2, and the letter Y was used as a probe in the experiment. There were two blocked conditions: related prime-target pairs (A–A) and unrelated prime-target pairs (B–A). Letters were displayed in uppercase, fixed width Batang font. To ensure distinct processing of primes and targets in the related condition, all primes were slightly smaller than targets fitting within a 3.8 × 3.8 cm space at the screen center, subtending a visual angle of 1.5°. Targets fit within a 4.5 × 4.5 cm space at the screen center, subtending a visual angle of 1.7°. All critical letters were seen twice as targets and twice as primes in each condition. Half of all critical trials (*N* = 92) were in the related condition (A–A) and half were present in the unrelated condition (B–A). Two different lists, each containing pseudorandomized stimulus pairs, were counterbalanced across participants.

#### Procedure

In this study, we examined how single English letters are processed by deaf and hearing readers. ERPs were recorded while participants performed a letter (probe) detection task. Participants were asked to press the button when the letter Y appeared either in the prime or target position. The probe appeared in 12% of all trials. Trials containing the probe were excluded from the analysis to avoid contamination of activity generated by the execution of a motor response. In the rest of the trials, participants passively monitored the letters without providing an overt response.

**Figure 1.**
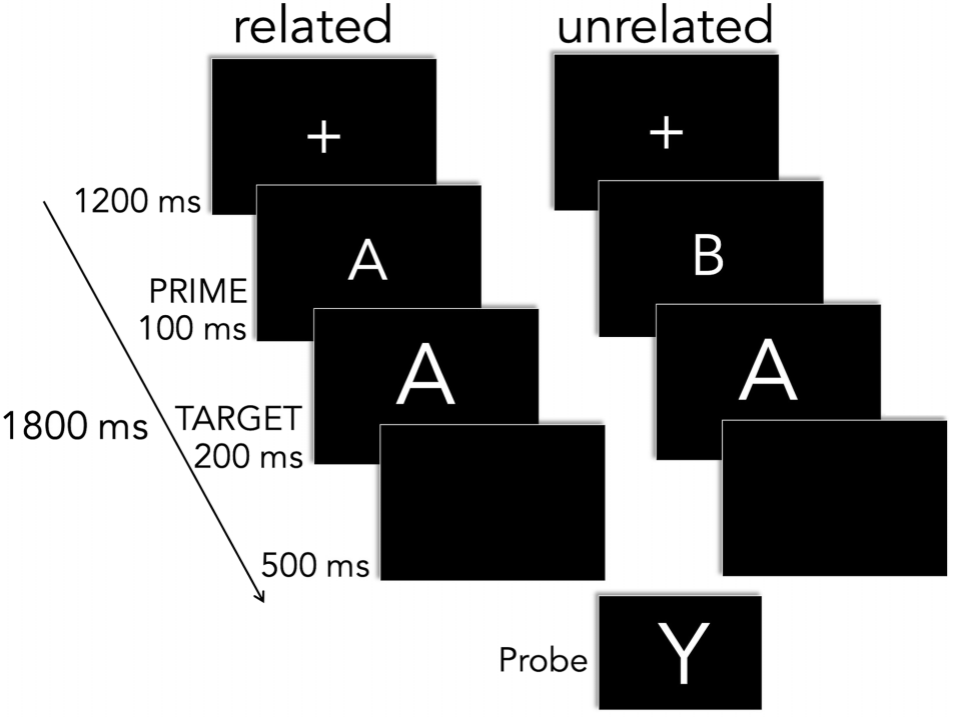
An example of a trial where English letter targets were preceded by related or unrelated letter primes. The probe, letter Y, appeared in either prime or target position in 12% of trials.

Stimuli were presented on a 24-inch ASUS VG248 monitor at a screen resolution of 1920 × 1080 pixels and a refresh rate of 100 Hz. The monitor was located approximately 145 cm (60 in) directly in front of the participant. Stimuli were displayed in high contrast as white letters on a black background. A trial consisted of a purple fixation cross in the middle of the screen (800 ms), during which the participant could blink. The purple fixation cross was replaced by a white fixation cross (400 ms) alerting the participant a trial was about to begin. After a blank screen of 200 ms, the prime appeared in the middle of the screen for 100 ms and was immediately followed by the target, displayed for 200 ms in the same location as the previous prime. The target was followed by a blank screen of 400 ms duration and finally the return of the purple fixation. Participants were given a brief rest break of about 2 min. approximately every 40 trials. An example of a trial where English letter targets were preceded by related or unrelated letter primes is shown in Figure 1.

#### EEG recording and analysis

Each participant sat in a comfortable chair in a darkened and sound attenuated room as an electroencephalogram (EEG) was recorded from 29 electrodes in an Electro-Cap (Electro-Cap International, 2023) using a left mastoid reference. Note that we also recorded EEG from the right mastoid with the left mastoid as reference. This allowed for the possibility of re-referencing all scalp data to the computed average of the two mastoids should there be evidence of differential activity at the right mastoid site. Careful inspection of the ERPs from the right mastoid in both experiments did not suggest such differences, so the data were not re-referenced. EEG was amplified with SynAmpsRT amplifiers (Compumedics Neuroscan, 2022) using a band pass of DC (direct current) to 100 Hz and was sampled continuously at 500 Hz. Offline, ERPs were time locked to target onset for each participant and priming condition using a 200 ms prestimulus baseline and low-pass filtered at 15 Hz. A loose electrode placed below the left eye was used in conjunction with recordings from FP1 to detect blink artifacts and another electrode on the outer canthus of the right eye was used to detect horizontal eye movement artifacts. Impedances were maintained below 2 kΩ for all electrode sites. Trials with artifacts during the baseline period or within 600 ms of target onset were excluded from analyses (3.9% of all trials).

**Figure 2.**
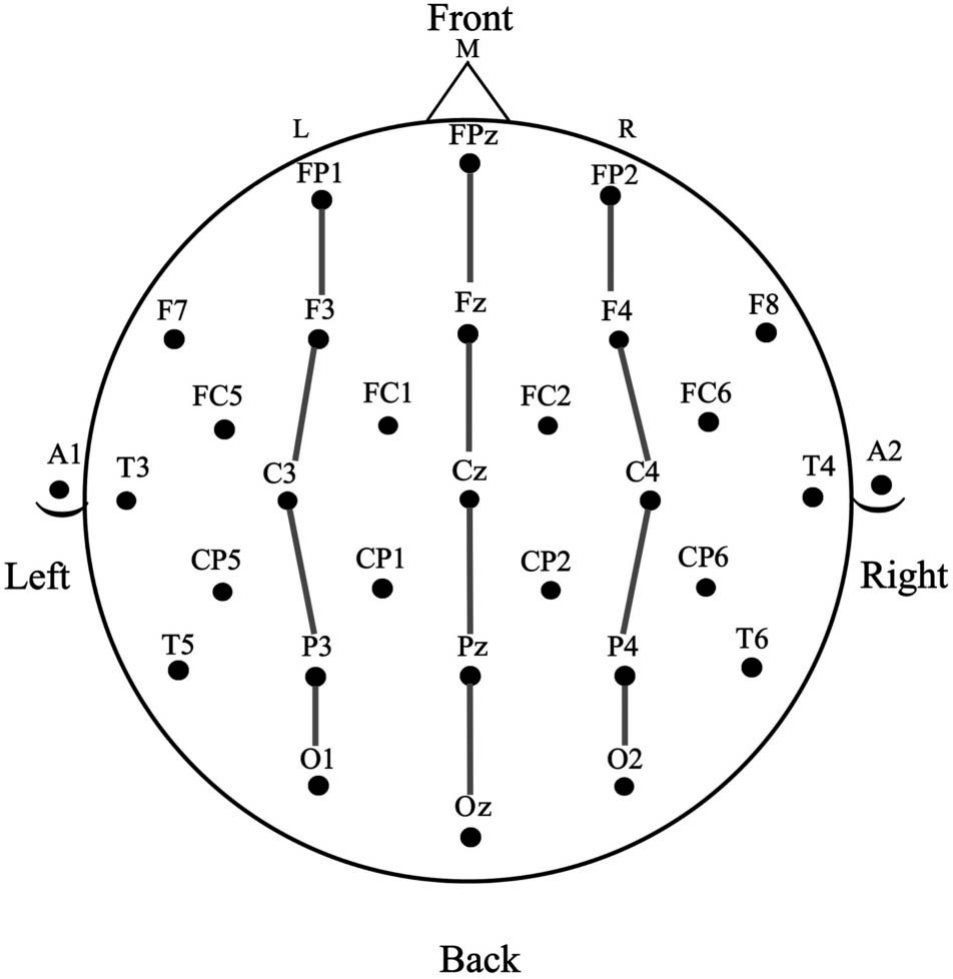
The modified 10–20 system electrode montage was used in this study. The sites used in the data analyses are indicated using enlarged circles. Electrode columns: laterality electrodes—left (L): FP1, F3, C3, P3, O1; midline (M): FPz, Fz, Cz, Pz, Oz; right (R): FP2, F4, C4, P4, O2. Anteriority electrodes—prefrontal: FP1, FPz, FP2; frontal: F3, Fz, F4; central: C3, Cz, C4; parietal: P3, Pz, P4; occipital: O1, Oz, O2. Mastoid electrodes: A1 (left), A2 (right).

A subset of 15 electrodes frequently used in the literature was selected for statistical analyses (see Figure 2). This electrode array is also similar to that used in Petit et al. (2006) but slightly reduced. The electrode array submitted to the ANOVAs was reduced compared to Petit et al. to lower the number of analyses of variance (ANOVAs) conducted. Instead of four columns of electrodes tested in four different ANOVAs, here the array is three columns, left, center, and right, with five electrodes from anterior sites to posterior sites submitted to one ANOVA.

To test for priming effects, ANOVAs with factors of Group (deaf, hearing), Relatedness (related, unrelated), and two distributional variables, Laterality (left, midline, right) and Anteriority (prefrontal, frontal, central, parietal, occipital) were applied on the mean amplitudes within two successive time windows. An early window (120–180 ms) was chosen based on visual inspection and its congruency with results reported by Petit et al. (2006) for the directionality of abstract letter recognition. Finally, as is typical in supraliminal priming studies, we also measured the amplitude of the N400 (300–500 ms). The Greenhouse-Geisser correction was used for all repeated-measures factors with greater than 1 degree of freedom in the numerator. (For ERP results from all 15 electrode sites included in the analyses below, see Figures S1–S3 in the supplementary materials available at https://osf.io/kzy3t/. For brevity, we included statistical findings that pertain to the questions and conclusions that we report in this article. For full statistical reports, see Tables S1–S3 in the supplementary materials at https://osf.io/kzy3t/).

### Results

Participants were successful at detecting probes (deaf hit rate: 98.6%; *SD* = 2.8%; hearing hit rate: 97.8%; *SD* = 4.2%) with no difference between groups, *F*(1, 46) = 0.66, *p* = 0.421. There was a marginal difference between hit rate for probe primes and probe targets, *F*(1, 46) = 3.87, *p* = 0.055) with greater accuracy for probe targets (99.4%, *SD* = 2.2%) compared to prime probes (98.4%, *SD* = 3.2%). There was no interaction between probe type or group, *F*(1, 46) = 0.32, *p* = 0.573. The false alarm rate for the deaf group was 0.6% and for the hearing group was 1.2% with a marginal difference between groups, *F*(1, 46) = 3.01, *p* = 0.089.

### ERP Results

**Figure 3.**
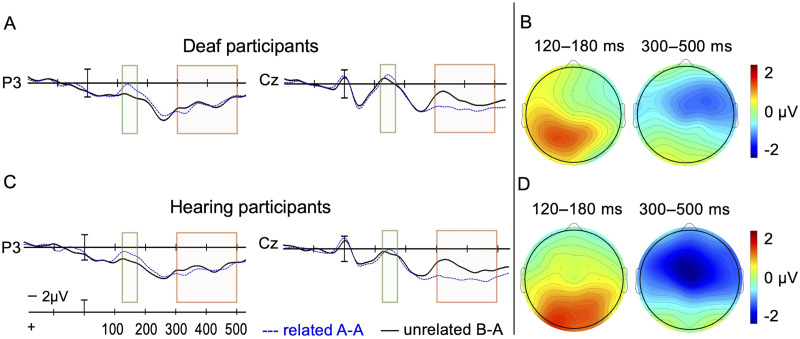
Event-related potential results from two representative electrode sites included in the analysis. (A) Deaf participants and (C) hearing participants. Voltage maps illustrate the lesser posterior positivity for related vs. unrelated pairs in the 120–180 ms time epoch, and greater negativity to unrelated vs. related pairs in the 300–500 ms time epoch (N400 window) for (B) the deaf participants and (D) the hearing participants across all 15 sites. Negative amplitude is plotted up.

As can be seen in Figure 3 deaf and hearing participants produced a similar pattern of early exogenous ERPs generated by a combination of the prime and target stimuli. Because of the close temporal proximity of these two events (100 ms) there is likely overlap in the exogenous ERP components generated by these two events, as has been reported in numerous previous ERP masked priming studies (see Grainger & Holcomb, 2009). However, because we carefully matched the two conditions for timing and type of pre-target events, any differences in target ERPs should be due to the relatedness manipulation. As can be seen in Figure 3, there is a clear early occipital positivity at 100 ms post-prime onset (P1) followed by a negativity at about 150 ms (N1). The occipital target P1 can also clearly be seen peaking at ∼100 ms post-target onset in both groups of participants. The effects of priming seem to be confined to portions of the post-target waves after 100 ms.

#### 120–180 ms epoch

There was a significant main effect of Group, *F*(1, 46) = 4.2, *p* = 0.047, suggesting that overall deaf participants exhibited more negative amplitude than hearing participants in this window. There were significant two-way Relatedness × Laterality, *F*(2, 92) = 5.6, *p* = 0.009, and Relatedness × Anteriority interactions, *F*(4, 184) = 4.3, *p* = 0.029, and a marginal effect of Relatedness, *F*(1, 46) = 2.8, *p* = 0.099, but no interaction between the Group and Relatedness was found, *p* = 0.847. The distribution of effects is shown in Figure 3 where the lesser positivity elicited by related targets was most apparent at electrodes in the left posterior quadrant (see Figure 3A and 3C, sites P3, Pz, O1, Oz). This priming effect appears to be distributed similarly for deaf and hearing participants, but because there was also a marginal three-way interaction of Group, Relatedness, and Laterality, *F*(2, 92) = 3.2, *p* = 0.081, the priming effect may be somewhat more left lateralized in the deaf participants (compare voltage maps in Figure 3B and 3D).

#### 300–500 ms epoch

There was a main effect of Relatedness, *F*(1, 46) = 5.47, *p* = 0.024, such that targets preceded by unrelated primes elicited larger negativity than targets preceded by related primes, but in this window, there was no main effect of Group, *p* = 0.119. There were also significant two-way Relatedness × Laterality, *F*(2, 92) = 4.2, *p* = 0.028, and Relatedness × Anteriority interactions, *F*(4, 184) = 8.0, *p* = 0.001. Although Figure 3 suggests a slightly larger N400 effect for the hearing group, an interaction between Group and Relatedness was not found, *p* = 0.336. Figure 3B and 3D voltage maps show that the greatest negativity to the unrelated targets relative to related targets was observed in the central (C3, Cz, C4) and central-anterior (F3, Fz, F4) sites with a slightly right-lateralized distribution. There were no other main effects or interactions (all *p*s ≥ 0.112). Figure 3 shows waveforms from two representative sites. (All 15 electrode sites are plotted in Figure S1, and the full ANOVA table can be found in Table S1, of the supplementary materials at https://osf.io/kzy3t/.)

### Discussion

Experiment 1 investigated single letter-to-letter priming effects in deaf and hearing readers. Specifically, we examined the influence of prime–target name consistency on ERPs generated by English letter targets in an unmasked priming paradigm. This allowed us to track the abstract levels of English letter processing during the early and late stages of letter recognition. The experiment yielded two main results. The first finding was that both deaf and hearing participants exhibited similar early letter-to-letter priming effects during the 120–180 ms time epoch, which manifested as a more positive ERP response to related than unrelated letter pairs. This relatively posterior priming effect of name consistency aligns with Petit et al. (2006, Figure 3B), who reported this effect as slightly later, between 220 and 300 ms (the red zone, or P260), and interpreted it as reflecting the abstract stage of letter recognition. Our results showed a similar though slightly earlier time course, perhaps due to the unmasked paradigm, and modest evidence for a more left lateralized scalp distribution in deaf compared to hearing readers.

Secondly, a priming effect for related letter pairs also occurred in the later epoch (300–500 ms) in both groups. Targets preceded by same-letter primes continued to be more positive-going than targets preceded by different-letter primes. This priming effect resembled a typical N400-like repetition effect, which tends to be associated with reduced processing effort for related (repeated) compared to unrelated stimuli. In single word studies, the N400 effect is typically interpreted as a measure of late word and/or semantic processing. Petit et al. (2006) did not report an N400 effect in their masked letter priming study. In our study, participants might have had to focus more intently on letter names because of the nature of the go/no-go task (i.e., on each trial, they had to assess whether the prime or target letter was the probe “Y”), while in the Petit et al. study participants performed a delayed keyboard response (i.e., they typed the target letter after each trial), which likely does not encourage letter name generation.

Alternatively, this N400-like effect might have occurred because of the use of supraliminal primes. Supraliminal primes might have encouraged an intentional comparison of prime and target letters, which in the case of different prime and target letters might have generated a mismatch negativity N400 (e.g., see Misra & Holcomb, 2003). Note that we did not analyze activity between the early and late time window because we did not manipulate letter case, in contrast with Petit et al. (2006). Regardless, we conclude that both deaf and hearing participants activated the featural information and abstract letter representations at similar stages of the time course of processing. In line with our prediction, the timing of visual letter recognition unfolds in a similar fashion and was not impacted by deafness or signing experience. The study demonstrated that both deaf and hearing readers rapidly access abstract letter representations and extended the results found by Petit et al. for hearing participants to unmasked (supraliminal) priming paradigm with both hearing and deaf participants. Future research could include participants with diverse reading levels to examine whether letter priming patterns might differ in less skilled readers. The study has also contributed evidence to the theoretical model of hierarchical letter processing congruent with results obtained using masked priming and ERPs with word and object stimuli (Eddy et al., 2006; Holcomb & Grainger, 2006). The results align with previous work indicating that in conditions where the prime can be consciously processed (e.g., 100 ms primes), the pattern of effects tends to be similar to what is found with shorter (50 ms) unconscious (masked) primes (Eddy et al., 2006).

## EXPERIMENT 2: PRIMING EFFECTS BETWEEN ASL FINGERSPELLING AND ENGLISH LETTERS

Experiment 2 aimed to establish whether there are differences between English letter processing and fingerspelling font processing in deaf signers. The main goal was to examine the extent to which English letters and fingerspelling fonts share abstract representations by comparing the ERP priming effects across the two orthographic systems in a group of deaf ASL–English bilinguals. Experiment 2A (English letter targets) examined letter priming by letters (within-domain) and by fingerspelling font primes (cross-domain). Experiment 2B (fingerspelling font targets) examined fingerspelling font priming by fingerspelling font (within-domain) and by English letter primes (cross-domain). Our secondary goal was to replicate the letter-to-letter priming results from Experiment 1. To reiterate our predictions, for Experiment 2A we expected that related letter pairs would elicit priming effects (i.e., less positivity) compared to unrelated pairs over the early ERP measurement epoch (120–180 ms), and thus replicate the letter-to-letter priming effects we found in Experiment 1. If fingerspelling fonts and letters share the same abstract representations, we expect to observe similar priming in this early window (120–180 ms) for fingerspelling font priming. Following a similar argument for Experiment 2A, if fingerspelling fonts and English letters share abstract representations then cross-domain priming in the same direction (less positivity for related pairs) would be expected during this early window. Because both primes and targets can be consciously processed with the timing of stimuli used here and following the results of Experiment 1, we also predicted within-domain priming in the later N400 epoch (300–500 ms) for both Experiments 2A and 2B.

### Method

#### Participants

Twenty-six adult severely to profoundly deaf bimodal bilinguals (ASL–English) were recruited to participate in Experiment 2. Two participants were excluded for high artifact rejection (>17% rejection in at least one condition). All participants reported having normal or corrected to normal vision, no history of neurological dysfunction or language disorders, were not taking any medications that would affect brain function, and provided written informed consent in accordance with the Institutional Review Board at San Diego State University, which approved the study. The remaining 24 participants (13 female; *M* age = 33 years, *SD* = 9) were either native ASL signers who were born into deaf signing families and exposed to ASL from birth (*N* = 10) or acquired ASL before age eight (*N* = 14; average age of ASL exposure = 36 months). All participants reported using ASL as their main form of communication and reported severe to profound hearing loss (dB loss ≥ 70 dB). Three participants were left-handed, and two had cochlear implants. Seventeen of these deaf participants also participated in Experiment 1. To minimize potential repetition effects, we ensured sufficient time (at least 6 months) had passed between testing sessions.

#### Stimuli

The stimuli were 23 single English letters displayed in uppercase fixed-width Batang font and 23 corresponding ASL fingerspelling fonts presented in the Handtext font (created by Joachim Lapiak; www.lapiakdesign.com/handtext/). For both types of letters, J, Z, and Y were excluded. J and Z were excluded because the production of these fingerspelling handshapes includes movement and thus cannot be fully represented in a still font. The letter Y was used as a probe.

**Figure 4.**
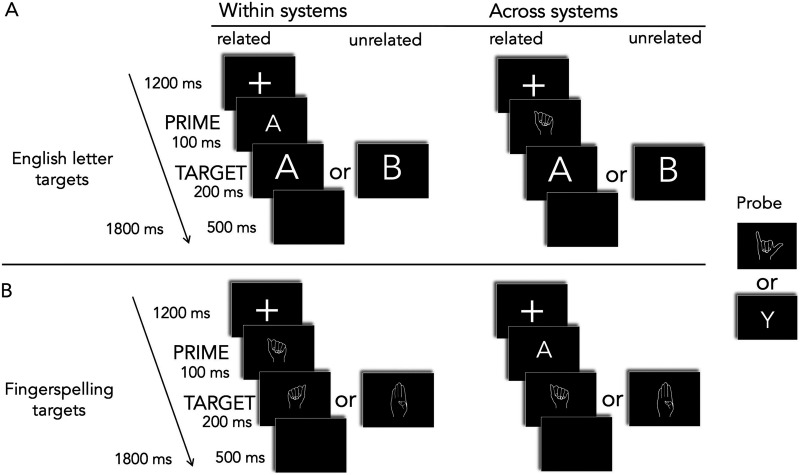
An example of related and unrelated trials. (A) Experiment 2A with English letter targets. (B) Experiment 2B with fingerspelling font targets. The probe, letter Y, appeared in either prime or target position in 12% of trials.

As in Experiment 1, to ensure distinct processing of the stimulus primes and targets in the related condition, all English letter primes were slightly smaller than the targets, fitting within a 3.8 × 3.8 cm space at the center of the screen, subtending a visual angle of 1.5°. The letter targets fit within a 4.5 × 4.5 cm space at the screen center, subtending a visual angle of 1.7°. All prime and target fingerspelling font stimuli were the same sizes, fitting within a 5 × 5 cm space at the screen center, subtending a visual angle of 1.9°. In the related (identical) handshape font pairs, primes and targets were always presented in reversed handedness. For the unrelated trials, half of the prime-target pairs were reversed in handedness and half were not, such that right- and left-handed fonts were shown an equal number of times. There were two types of priming, within and across domains, which we exemplified in Figure 4.

##### Experiment 2A—English letter targets.

All 23 critical English letter stimuli were seen twice as targets in each of four conditions: within the orthographic system (within-domain) related (e – E) and unrelated (x – E), across the orthographic systems (cross-domain) related (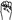 – E) and unrelated (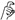 – E); thus, each condition included 46 trials, and there was a total of 184 critical trials. Two different lists in pseudorandomized order of stimulus presentation were counterbalanced across participants.

##### Experiment 2B—Fingerspelling targets.

All 23 critical fingerspelling font stimuli were seen twice as targets in each of four conditions: within-domain related (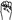 – 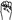) and unrelated (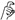 – 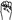), cross-domain related (e – 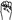) and unrelated (x – 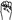). Thus, each condition has 46 trials for a total of 184 critical trials. Again, two different lists in pseudorandomized order of stimulus presentation were counterbalanced across participants. For both parts of the experiment, the participants’ task was to press to all Ys (either a letter or font) in the prime or target position, of which there were 50 (27% of all trials).

#### Procedure

The procedure was the same as Experiment 1. In addition, prior to the experiment, participants were familiarized with the fingerspelling font stimuli. During this familiarization, each handshape font stimulus (in either right-handed or left-handed) was displayed individually on the screen for 200 ms. Participants typed the corresponding English letter on the keyboard. As soon as they recorded their response, the next handshape font ensued. Participants performed at or near ceiling and did not report any difficulties recognizing the left- or right-hand fonts. The participants also completed the Spelling Recognition Test (Andrews & Hersch, 2010; *M* = 71, *SD* = 9, 81% accuracy), and additionally completed a test of fingerspelling reproduction to assess fingerspelling skills, the FS Reproduction Test (The VL2 FS Test; Morere & Allen, 2012). In this test, participants viewed video clips of naturalistically produced fingerspelled words (*N* = 45) and pseudowords (*N* = 25) that were taken from the Spelling and Spelling of Sounds subtests of the Woodcock-Johnson III Tests of Achievement (Woodcock et al., 2001). After each clip, they were required to repeat (i.e., fingerspell) the item they had just seen. The participants performed with 82% accuracy on this test, indicating they were skilled at fingerspelling.

#### EEG recording and analysis

Electrode placement, recording, referencing, amplification, sampling rate, time locking, baselining, and artifact rejection were the same as in Experiment 1. A total of 2.3% of all trials were excluded from analyses due to artifacts. The same subset of 15 electrodes as in Experiment 1 was used for statistical analyses (see Figure 2).

The analyses were carried out separately for Experiments 2A (English letter targets) and 2B (fingerspelling font targets); we conducted ANOVAs with Orthographic Domain (within, across), Relatedness (related, unrelated), Laterality (left, midline, right), and Anteriority (polar-frontal, frontal, central, parietal, occipital) with average amplitude as the dependent variable within the same windows as in Experiment 1, 120–180 ms and 300–500 ms epochs. As we were only interested in priming effects, we reported main effects and interactions pertaining to the relatedness status between primes and targets for brevity. We omitted all other not-of-interest or non-significant main effects and interactions from the statistical report.

### Results

#### Experiment 2A—English letter targets

Participants were successful at detecting probes with a near-ceiling hit rate of 97.8% (*SD* = 2.8%). The false alarm rate was 1.1%. Participants were significantly better at detecting probes in the target position (99.1%, *SD* = 0.02) compared to the prime position (94.7%, *SD* = 0.08), *F*(1, 23) = 7.4, *p* = 0.012.

##### 120–180 ms epoch.

**Figure 5.**
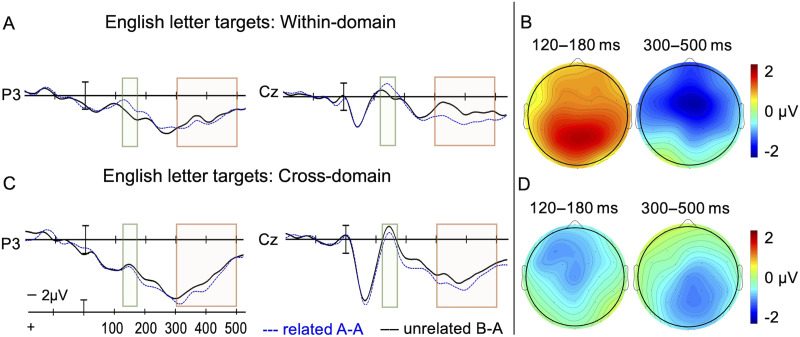
English letter targets. (A) ERP results from two representative electrode sites included in the analysis when letter targets were preceded by letter primes (within-domain). (B) Voltage map illustrating the lesser posterior positivity for related vs. unrelated pairs in the 120–180 ms time epoch, and greater negativity to unrelated vs. related pairs in the 300–500 ms time epoch (within-domain) for all 15 sites. (C) ERP results from two electrode sites included in the analysis when letter targets were preceded by fingerspelling font primes (cross-domain). (D) Voltage map illustrating a non-significant difference between waveforms in the 120–180 ms time epoch (*p* = 0.093), and a significant priming effect characterized by greater posterior negativity to unrelated vs. related pairs in the 300–500 ms epoch (cross-domain). Voltage maps show amplitude to unrelated minus related targets in the cross-domain condition. Negative amplitude is plotted up.

There was a main effect of Domain, *F*(1, 23) = 16.0, *p* < 0.001, and a two-way Domain × Relatedness interaction, *F*(1, 23) = 10.5, *p* = 0.004. A follow-up analysis for within-domain priming of English letter targets (plotted in Figure 5A and 5B) confirmed that English letter targets preceded by related English letter primes elicited less positive amplitude than unrelated pairs aligning with results in Experiment 1 (main effect of Relatedness, *F*(1, 23) = 7.0, *p* = 0.014). However, no such priming was observed for the reverse condition. A follow-up analysis for cross-domain priming of English letter targets preceded by fingerspelling font primes showed no significant difference between related and unrelated pairs (no main effect of Relatedness, *p* = 0.093). Non-significant main effects and interactions were all *p*s ≥ 0.144.

##### 300–500 ms epoch.

There was a main effect of Domain, *F*(1, 23) = 17.4, *p* < 0.001, and a main effect of Relatedness, *F*(1, 23) = 6, *p* < 0.022. There was a three-way Domain × Relatedness × Anteriority interaction, *F*(4, 92) = 5.66, *p* = 0.006. A follow-up analysis for within-domain priming revealed a main effect of Relatedness, *F*(1, 23) = 4.5, *p* = 0.045, and a Relatedness × Anteriority interaction for English letters, *F*(4, 92) = 3.9, *p* = 0.020, suggesting that letter targets preceded by related letter primes elicited smaller N400-like negativity than unrelated pairs and that the priming effects were distributed predominantly across anterior and central sites (Figure 5B). Cross-domain priming produced a significant Relatedness × Anteriority interaction, *F*(4, 92) = 4.4, *p* = 0.021, suggesting that English letter targets preceded by unrelated fingerspelling font primes elicited greater negativity than related font primes with a more posterior distribution (Figure 5D). (All 15 electrode sites are plotted in Figures S2 in the supplementary materials available at https://osf.io/kzy3t/. The full ANOVA tables [Tables S2–S4] can be found in the supplementary materials at https://osf.io/kzy3t/.)

#### Experiment 2B—Fingerspelling font targets

Participants were successful at detecting probes (either the letter Y or fingerspelling font Y) with a hit rate of 98.4% (*SD* = 2.3%). The false alarm rate was 1.2%. Participants were significantly better at detecting probes (either the letter Y or fingerspelling font Y) in the target position (99.3%, *SD* = 0.02) compared to the prime position (96.5%, *SD* = 0.05), *F*(1, 23) = 8.2, *p* = 0.009.

##### 120–180 ms epoch.

There was a main effect of Domain, *F*(1, 23) = 18.5, *p* < 0.001, that is, fingerspelling font primes and targets generated more positive amplitude than English letter primes, regardless of whether the pairs were related. No other main effects or interactions reached significance in this epoch (all *p*s > 0.102).

##### 300–500 ms epoch.

**Figure 6.**
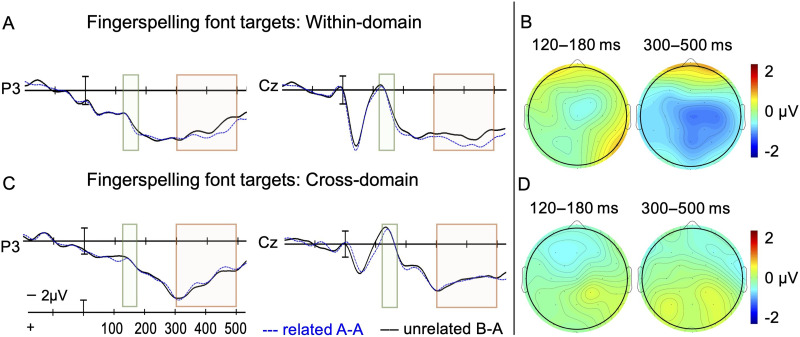
Fingerspelling font targets. (A) ERP results from two representative electrode sites included in the analysis when fingerspelling font targets were preceded by fingerspelling font primes (within-domain). (B) Voltage map illustrating the greater posterior positivity for related vs. unrelated pairs in the 120–180 ms time epoch, and greater negativity to related vs. unrelated pairs in the 300–500 ms time epoch (within-domain) in all 15 sites. (C) ERP results from two electrode sites included in the analysis when fingerspelling font targets were preceded by English letter primes (cross-domain). (D) Voltage map illustrating a lack of priming effects in the 120–180 ms time epoch, and greater posterior negativity to related vs. unrelated pairs in the 300–500 ms time epoch (cross-domain) in all 15 sites. Voltage maps show amplitude to unrelated minus related targets in the cross-domain condition. Negative amplitude is plotted up.

There was a three-way interaction between Domain, Relatedness, and Anteriority, *F*(4, 92) = 5.8, *p* = 0.010. A post hoc follow-up analysis for the within-domain condition revealed a significant Relatedness × Anteriority interaction, *F*(4, 92) = 5.9, *p* < 0.013, suggesting that fingerspelling font targets preceded by related fingerspelling fonts elicited smaller, N400-like negativity than unrelated pairs. This priming effect was most apparent in the central-posterior regions with a slightly right-sided distribution (Figure 6A and 6B). Cross-domain priming, however, produced no main effect of Relatedness (*p* = 0.382) or interactions with Relatedness (all *p*s ≥ 0.281), suggesting a lack of priming effect between letter primes and fingerspelling font targets in this time window (Figure 6C and 6D). Figure 6 shows waveforms from two representative sites. (All 15 electrode sites are plotted in Figures S3 in the supplementary materials available at https://osf.io/kzy3t/. The full ANOVA tables [Tables S5–S7] can be found in the supplementary materials at https://osf.io/kzy3t/.)

### Discussion

Experiment 2A examined English letter priming by English letters (within-domain) and by ASL fingerspelling font primes (cross-domain) in deaf ASL–English bilinguals. Experiment 2B examined handshape font priming by handshape fonts (within-domain) and by English letter primes (cross-domain).

There were four main findings. First, we replicated the expected letter-to-letter abstract identity priming effect we observed in Experiment 1 for deaf and hearing participants. This abstract letter priming effect onset earlier, around 120 ms, compared to the same effect reported by Petit et al. (2006) for hearing participants, which onset around 220 ms. Specifically, related English letter pairs attenuated the positivity of the early ERP responses compared to unrelated letter pairs about 120 ms after the target onset. This letter-to-letter priming effect again continued to be present in the later epoch where the reverse polarity (N400-like) may reflect access to letter names. This effect was characterized by central-anterior scalp distribution such that the stimulus relatedness attenuated the negativity of the fourth peak. Therefore, both Experiment 1 and Experiment 2A provided convincing evidence that deaf people process single English letters similarly to hearing people and that knowledge of the ASL fingerspelling system and/or deafness do not fundamentally alter visual, single letter processing. While differences in orthographic processing between deaf and hearing readers were previously found (Emmorey et al., 2017; Glezer et al., 2018; Gutierrez-Sigut et al., 2022; Sehyr et al., 2020), these differences were observed at a whole word level, not at the single character level.

The second main result was that although we found cross-domain priming, these priming effects were asymmetric (unidirectional). That is, fingerspelling fonts primed English letters (Figure 5C and 5D), but English letters did not prime fingerspelling fonts (Figure 6C and 6D). Further, fingerspelling primed letters only in the later epoch (300–500 ms): English letters preceded by related fingerspelling fonts elicited less negativity in the central-right posterior sites than unrelated pairs resulting in an N400-like priming effect. In the early 120–180 ms window, the negative difference in brainwaves between related and unrelated pairs did not reach significance (Figure 5C and 5D).

The absence of a fingerspelling-to-letter priming effect in the early window can be attributed to the different nature of the stimulus types. The fingerspelling fonts represent dynamic handshapes that can be recognized as fonts but that signers do not typically read, unlike letters that represent speech sounds. Visually, the fingerspelling fonts and letters have little overlap. It is perhaps less surprising that there was no modulation of the P150 component which tends to index low-level feature analysis. Priming effects in the early time epochs tend to reflect activity in neural processes that are sensitive to the physical changes in the stimulus, such as changes in size and orientation (Petit et al., 2006). The lack of early priming thus suggests that representations associated with fingerspelling fonts and letters are not part of a shared orthographic system. However, the attenuated negativity downstream, the N400-like priming effect, could reflect a process of spreading activation among the lexical-semantic representations of the fingerspelling fonts (handshape letter names) and English letter names. The supraliminal nature of the prime display may have encouraged the “naming” of the handshape fonts. These names could then prime, or co-activate, the English letter names and subsequently lead to lexical-semantic priming for identical letter names. However, no priming was found when fingerspelling font targets were preceded by English letters. That is, fingerspelling fonts did not benefit from preceding related letter primes. Letter primes may not activate lexical names as the fingerspelling fonts did because letters enjoy more automatic coding for rapid word assembly, unlike fingerspelling fonts. Nevertheless, these asymmetrical priming effects were still somewhat surprising and suggest that the nature of spreading activation among fingerspelling and letter representations is asymmetrical (unidirectional).

One explanation is that asymmetric priming may be related to language dominance. Although all participants in the present study were skilled English spellers as assessed by the spelling test, they indicated that ASL was their dominant and preferred language of communication. In fact, many deaf children from signing homes know fingerspelling before they learn orthography and reading in school (Padden & Le Master, 1985). Fingerspelling is often used along with reading instruction to teach deaf children about letters and words, which may reinforce the link between fingerspelling handshapes and orthography. Fingerspelling—part of deaf participants’ dominant or first language (L1)—may thus activate stronger representations than the English letters (participants’ second language, L2), resulting in priming effects from fingerspelling to English letters but no or weak priming effects from letters to fingerspelling. Asymmetry in cross-language priming has been regularly reported in the bilingual literature, typically with stronger priming effects when the prime was in the participants’ L1 and the target was in their L2 (Keatley et al., 1994; Midgley et al., 2009; Schoonbaert et al., 2011). The asymmetry suggests that the representations of letters expressed in different orthographic systems are stored in separate language systems and perhaps rehearsed and represented differently in memory. These representations may be interconnected via meaning-integration processes or one-to-one links between translation or lexical-semantic equivalents. More research needs to be done to establish whether priming strength is modulated by ASL and/or English proficiency.

A somewhat similar asymmetry in short-term memory for fingerspelling and print representations was observed in a serial recall study that presented printed and (dynamic) fingerspelled words. Sehyr et al. (2017) found that adult skilled deaf readers utilized speech-based phonological coding to recall lists of printed and fingerspelled words from short-term memory, a result that lends support to a relationship between fingerspelling and print (Sehyr et al., 2017). But the deaf readers did not re-code fingerspelled or printed words into a manual-based (fingerspelled) representation—a manual similarity effect was not observed for either word type. The authors suggested that this asymmetry may arise due to demands on memory, reflecting the limited capacity of short-term memory for fingerspelling because fingerspelling takes longer to articulate than speech. As a result, fingerspelled words may be rapidly re-coded into English-based phonology because a speech-based code lends itself better for rehearsal of information in the temporally structured short-term rehearsal loop. While short-term memory plays an important role in reading and fingerspelling recognition, it may be relatively inefficient for deaf readers to re-code English letters into a manual-based fingerspelling code during reading. Our result echoes this asymmetry. Fingerspelling is not stored (rehearsed) in the manual form but is re-coded into a speech-based form which may co-activate English letter representations. The fingerspelling to English letter priming effect supported this. The reverse did not occur—printed words were not re-coded as fingerspelling, so we did not observe letter-to-fingerspelling priming. Readers develop highly specialized rapid processing for reading letters attributable to experience with reading. Such specialized processing for fingerspelling fonts does not occur since no one regularly reads fingerspelling fonts.

Another plausible explanation for the asymmetric priming effects is that the fingerspelling fonts may have been processed as objects or pictures. ERP patterns reflecting picture repetition priming effects are different than those reflecting word repetition priming effects (Eddy et al., 2006; Zhang et al., 1997). It is possible that 300 ms after stimulus onset, the fingerspelling fonts activated some type of lexical-semantic representations of handshapes in ASL. In a way, the fingerspelling fonts represent the names of English letters—that is, the Y handshape is the lexical sign for the letter Y in ASL. Additionally, the fingerspelling fonts also represent handshapes that occur in signs that are initialized; for example, the sign for “yellow” contains the same Y handshape that is used to fingerspell the letter Y. Thus, handshape fonts could have activated lexical-semantic representations of letter names, which might have led to priming effects between related pairs. As mentioned earlier, priming effects in the opposite direction (from letters to fingerspelling) did not occur because letter processing is highly specialized, automatic, and connected to the higher-level word processing during reading. As such, single letters alone might not evoke semantic representations of letter names sufficiently to prime fingerspelling fonts. This explanation accounts for the two remaining results.

As Figure 6B shows, our third main finding was that fingerspelling primed fingerspelling but in the later time epoch only. Recall that we included an equal number of left-handed and right-handed orientations of the fingerspelling fonts and that identical fingerspelling prime-target pairs were always presented in reversed orientation to perceptually separate the unmasked primes from targets. It is thus possible that early priming between fingerspelling fonts was reduced due to the orientation reversal, because early visual recognition is sensitive to changes in physical properties including orientation. We did, however, observe a significant priming effect in the later N400 window where priming is no longer sensitive to orientation change. One possible explanation for this result is that participants mapped the fingerspelling fonts onto abstract, orientation-invariant representations. This result aligns with results from visual object priming. Schendan and Kutas (2003) reported that priming effects were greater for objects repeated with the same view than with a different view, whereas later time windows (400–700 ms) showed repetition effects with greater generalization across views. To conclude, similarly to objects, early processing of fingerspelling fonts may be orientation-dependent, but later processing of fingerspelling appears to be orientation-invariant. Future work could compare priming effects between fingerspelling fonts presented in the same versus reverse orientation perhaps in a masked paradigm.

Finally, we observed that when fingerspelling fonts and English letters were intermixed within the experimental block, there was a more centralized (symmetrical) distribution of activity related to visual recognition than when only letters were presented in the block. That is, the letter-to-letter priming effects were distributed more centrally in the occipital sites (Figure 5B and 6B) when fingerspelling fonts were present (Experiment 2), but when fingerspelling fonts were not included in the task both early and late priming effects for English letters were more lateralized (Experiment 1); early effects were lateralized to the left temporal-occipital sites while late effects were lateralized to the right frontal sites (Figure 3B). To explain this pattern, we again consider the possibility that the fingerspelling fonts were processed in an object-like manner. Visual object priming effects typically occur as early as 100 ms and tend to be bilaterally distributed in central and posterior regions (Eddy et al., 2006; Henson et al., 2004; Holcomb & Grainger, 2006; Li et al., 2019; Schendan & Kutas, 2003). Visual word priming effects typically also emerge as early as 100–150 ms but tend to be lateralized to left hemisphere occipital/temporal regions (Maurer et al., 2005, 2008; Wong et al., 2005). If fonts activate non-orthographic object-like representations, this could shift the activity related to fingerspelling font recognition toward central regions. The processing of the distinct stimuli may thus modulate the demand on attentional resources and alter the distribution of neural activity in visual recognition.

To summarize the findings, deaf and hearing readers process English letters similarly, suggesting that knowledge of fingerspelling and/or deafness do not alter visual letter processing. This result extended previous findings for hearing readers (e.g., Petit et al., 2006) to supraliminal (unmasked) letter presentations. Second, fingerspelling fonts primed both fingerspelling fonts and English letters, but English letters did not prime fingerspelling fonts, indicating a priming asymmetry between letters and fingerspelling fonts. We also found an N400-like priming effect when the primes were fingerspelling fonts, which might have reflected strategic access to the lexical names of letters. Finally, when fingerspelling fonts were mixed in the task, the topographic distribution of the priming effects for letters was more centralized compared to a more lateralized distribution when the task only included letters, pointing to differences in attentional recruitment of networks responsible for the processing of the two stimulus categories. The studies suggest that deaf ASL–English bilinguals process English letters and ASL fingerspelling differently, and that the two systems may have distinct neural representations. However, the fact that fingerspelling fonts can prime English letters suggests that the two orthographies may share abstract representations to some extent.

These results are broadly consistent with a two-stage hierarchical model of visual object recognition that consists of a basic level of visual feature processing (around 100 ms after stimulus onset) followed by a second stage that involves item-specific identification processes—the letter stage (see McClelland & Rumelhart, 1981, for a generic interactive activation model of letter perception). Our results are not compatible with claims of a universal, cross-alphabetic hierarchical account of letter processing (e.g., see Carreiras et al., 2013). Unlike other letter representations, fingerspelling handshapes are dynamic productions that can be recognized as static line drawings or fonts; however, the recognition of static fingerspelling fonts does not appear to be highly automatized. Further research is needed to confirm that these conclusions generalize to dynamic fingerspelling presentations.

Understanding the relationship between fingerspelling and print has relevance for theories of orthographic and visual recognition and could help explain the differences in hemispheric lateralization to word recognition in deaf readers (Emmorey et al., 2017; Sehyr et al., 2020). The differences in print and fingerspelling processing can reveal how orthographic systems that represent the sounds of language (directly or indirectly) are mentally represented. A better characterization of the neurobiological nature of fingerspelling and its relationship to print can contribute to optimized reading instruction strategies for deaf students.

## ACKNOWLEDGMENTS

We would like to thank Cindy O’Grady Farnady, Jamie Renna, Stephanie Osmond, and Emily Akers for their assistance on this project, and all deaf and hearing participants.

## FUNDING INFORMATION

Karen Emmorey, National Institute on Deafness and Other Communication Disorders (https://dx.doi.org/10.13039/100000055), Award ID: R01 DC014246.

## AUTHOR CONTRIBUTIONS

**Zed Sevcikova Sehyr**: Conceptualization: Lead; Data curation: Equal; Formal analysis: Equal; Investigation: Equal; Methodology: Equal; Supervision: Equal; Visualization: Lead; Writing—original draft: Lead; Writing—review & editing: Lead. **Katherine J. Midgley**: Conceptualization: Lead; Data curation: Equal; Formal analysis: Lead; Investigation: Equal; Methodology: Equal; Project administration: Lead; Resources: Equal; Supervision: Equal; Visualization: Lead; Writing—original draft: Lead; Writing—review & editing: Equal. **Karen Emmorey**: Conceptualization: Lead; Funding acquisition: Lead; Investigation: Lead; Resources: Equal; Supervision: Equal; Writing—original draft: Lead. Writing—review & editing: Equal. **Phillip J. Holcomb**: Conceptualization: Equal; Data curation: Equal; Formal analysis: Equal; Investigation: Equal; Methodology: Equal; Resources: Lead; Software: Lead; Writing—original draft: Equal.

## DATA AVAILABILITY STATEMENT

The processing, filtering, averaging, and analyzing of the data were accomplished by a suite of dedicated in-house software developed by the NeuroCognition Lab at San Diego State University. The data and results are available at https://osf.io/kzy3t/.

## TECHNICAL TERMS

N400: The N400 wave is an event-related brain potential (ERP) measured using electroencephalography (EEG). N400 refers to a negativity peaking at about 400 ms after stimulus (i.e., written word) onset. It has been used to investigate semantic processing.
American Sign Language (ASL) fingerspelling: A manual, non-written symbol system used by ASL signers in which each letter of an alphabet is represented by a distinct handshape. Fingerspelling handshapes are formed and articulated sequentially to represent words or letter sequences from a spoken language (e.g., English).
Supraliminal priming: A type of priming where stimuli, for example, the prime or target words, can be perceived consciously by the participant. Although participants are aware of the stimuli, they are generally not aware of their influence on them.
